# Grassland versus forest dwelling rodents as indicators of environmental contamination with the zoonotic nematode *Toxocara* spp.

**DOI:** 10.1038/s41598-022-23891-6

**Published:** 2023-01-10

**Authors:** Martyna Krupińska, Daniela Antolová, Katarzyna Tołkacz, Klaudiusz Szczepaniak, Aneta Strachecka, Aleksander Goll, Joanna Nowicka, Karolina Baranowicz, Anna Bajer, Jerzy M. Behnke, Maciej Grzybek

**Affiliations:** 1 Department of Tropical Parasitology, Medical University of Gdansk, Powstania Styczniowego 9B, 81-519 Gdynia, Poland; 2 Institute of Parasitology of SAS, Košice, Slovakia; 3 University of Warsaw, Warsaw, Poland; 4 Institute of Biochemistry and Biophysics, Polish Academy of Science, Warsaw, Poland; 5 University of Life Sciences in Lublin, Lublin, Poland; 6 University of Nottingham, Nottingham, UK

**Keywords:** Ecological epidemiology, Grassland ecology, Microbial ecology

## Abstract

Small mammals are suspected of contributing to the dissemination of *Toxocara canis* and helping with the parasite survival during periods when there is a temporary absence of suitable definitive hosts. While the primary aim of the current study was the assessment of seroprevalence of *Toxocara* spp. infections in wild rodents in Poland, we also explored the role of intrinsic (sex, age) and extrinsic factors (study site) influencing dynamics of this infection to ascertain whether grassland versus forest rodents play a greater role as indicators of environmental contamination with *T. canis*. We trapped 577 rodents belonging to four species (*Myodes glareolus, Microtus arvalis, Microtus agrestis, Alexandromys oeconomus*) in north-eastern Poland. Blood was collected during the parasitological examination, and serum was frozen at − 80 °C until further analyses. A bespoke enzyme-linked immunosorbent assay was used to detect antibodies against *Toxocara* spp*.* We found *Toxocara* spp*.* antibodies in the sera of all four rodent species with an overall seroprevalence of 2.8% [1.9–4.1%]. There was a significant difference in seroprevalence between vole species, with the grassland species (*M. arvalis*, *M. agrestis* and *A. oeconomus)* showing a 16-fold higher seroprevalence (15.7% [8.7–25.9%]) than the forest-dwelling *M. glareolus* (0.98% [0.5–1.8%]). We hypothesise that the seroprevalence of *Toxocara* spp. differs between forest and grassland rodents because of the higher contamination of grasslands by domestic dogs and wild canids. Our results underline the need for wide biomonitoring of both types of ecosystems to assess the role of rodents as indicators of environmental contamination with zoonotic pathogens.

## Introduction

Human toxocariasis is one of the most widespread helminthic zoonoses globally^1^. According to the Centers for Disease Control and Prevention (CDC), toxocariasis is one of the six most important neglected parasitic infections in the United States^2^. Nevertheless, there are still many unknowns concerning *Toxocara* spp., including sources of infection and modes of transmission^3^.

*Toxocara canis* is a cosmopolitan nematode parasite of carnivores, notably canids, both wild and domestic, which act as definitive hosts^4^. Non-invasive unembryonated *T. canis* eggs are shed in large numbers in canine faeces^5^, and after several weeks, under appropriate environmental conditions, eggs can develop into an embryonated stage that serves as a source of infection for definitive and paratenic hosts^6,7^. Many different hosts can act as paratenic hosts, including humans, pigs, avian species and rodents^8^. Eggs consumed by paratenic hosts cannot develop further into the adult stage, but infective larvae can persist in host tissue for an extended time, constituting a reservoir of *T. canis* for canids^9^. The life cycle is completed when prey infected with arrested tissue larvae is eaten subsequently by a definitive host^10^. Small mammals are suspected of contributing to the dissemination of *T. canis* and helping with the survival of the parasite, especially during periods when there is a temporary absence of suitable definitive hosts. They can also play a role as an indicator of environmental contamination with *Toxocara*^11^*.*

Human infections are primarily associated with oral ingestion of the embryonated eggs of *T. canis* contaminating food items such as salad crops and vegetables or through geophagy. The possibility of transmission of *T. canis* eggs through dogs’ hair has been explored also, but research suggests that it is unlikely to be of major epidemiological significance^3^. The persistence of larvae in human tissues can cause several clinical symptoms classified into four^12^. The most commonly recognised are visceral larva migrans (VLM), usually diagnosed in young children, caused by larval migration through major organs such as the liver or lungs and ocular larva migrans (OLM), typically found in children and young adults, with pathological effects restricted to the eye and the optic nerve^13,14^. *T. canis* larvae can also invade the central nervous system resulting in neurotoxocariasis (NT) or cause non-specific symptoms, reflecting covert toxocariasis^15,16^. Parks, playgrounds and backyards constitute a frequent source of infection, especially for children playing in sandpits. Recent estimates show that seroprevalence of *Toxocara* spp. varies from 10% in the general population of Europe to 37.7% in that of Africa^17^. Between 1992 and 2012, 1022 cases of toxocariasis were recorded in Poland^18^.

There is now increasing interest in understanding the fine details of the transmission of pathogens and notably the different variables that might influence infection dynamics. Among these, extrinsic factors such as geographic location and time^19–23^ and intrinsic factors, including host sex, genetics, age, social and reproductive status^24–31^ are likely to play crucial, but varying roles in host susceptibility to different pathogens, and their persistence in both the short and long-term host populations. Hence, a comprehensive understanding of pathogen dynamics in their wildlife reservoirs is desirable, aiming to improve our appreciation of the epidemiology of these diseases in their wild reservoirs and in humans^32,33^. Such data are essential for informed decision-making on measures for preventing and controlling relevant pathogens^34–37^. While the primary aim of the current study was the assessment of seroprevalence of *Toxocara* spp. infections in wild rodents in Poland, we also explored the role of intrinsic (sex, age) and extrinsic factors (study site) influencing the dynamics of this infection.

## Material and methods

### Study sites and collection of rodents

**Figure 1:**
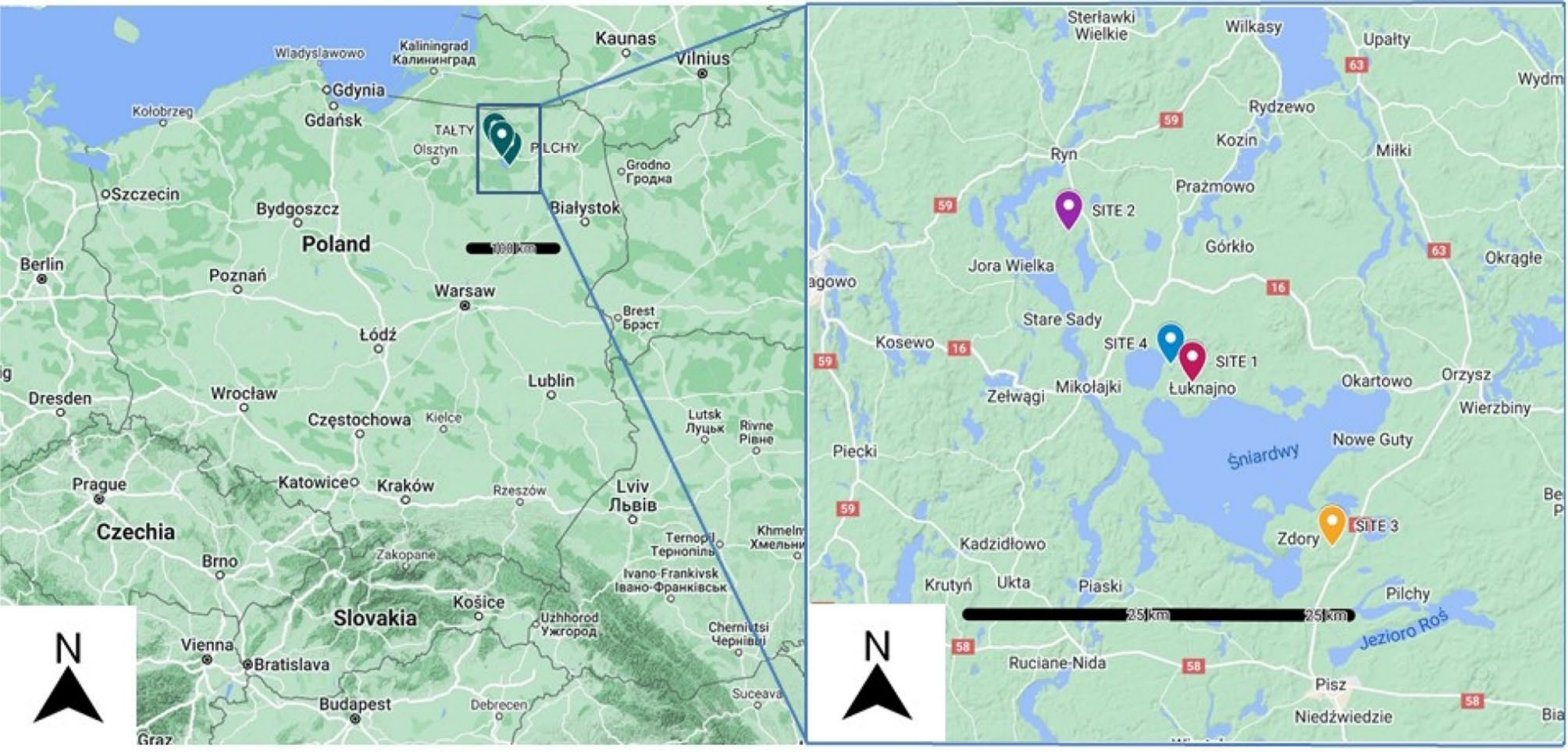
Map showing study sites located in north-eastern Poland. Study sites are located within Mazury Lake District. Site 1—Urwitałt forest; Site 2—Tałty forest, Site 3—Pilchy forest; Site 4—Urwitałt open grasslands. Black bars indicates the distance. (Map data, Google Maps 2022. https://maps.google.com).

Trapping of bank voles was completed at three trapping session in 2002, 2006 and 2010^27,38–40^. Trapping of grassland rodents were conducted in summer, 2013–2014. The study sites, comprehensively described in our earlier papers^27,38–40^, were located in the Mazury Lake District region in the north-eastern (NE) corner of Poland, in close proximity to towns Mikołaki, Ryn, Pisz and Śniardwy lake. Bank voles were collected from three mixed forests within 10 km one from another, separated by lakes, canals and rivers, marked on the map as Site 1 (N 53° 48.153, EO 21° 39.784), Site 2 (N 53° 53.644, EO 21° 33.049) and Site 3 (N 53° 42.228, EO 21° 48.499). Field, common and root voles were trapped in open grasslands with diverse scrub and tall grass vegetation marked on the map as Site 4 (N 53° 81.483, EO 21° 65.25) (Fig. 1). Rodents were caught in wooden traps. Methods for trapping, sampling and processing rodents have been thoroughly described^39,41–43^. Three age categories were established as described earlier using principal components analysis of a range of morphological measures, including body weight and dried eye lens weight^40,44^. Age class 1 voles were immature juveniles, age class 2 voles were primarily young adults and age class 3 were breeding older animals^27^.

Blood samples were collected directly from the heart using a sterile 1.5 mL syringe immediately after death from over-exposure to anaesthetic, and blood was allowed to clot at room temperature. After separating the blood clot, samples were centrifuged at 3.350 g for 10 min using a MPW High-Speed Brushless Centrifuge. Serum was collected and stored at – 80 °C until samples were analysed on completion of the fieldwork.

### Serology

An enzyme-linked immunosorbent assay (ELISA) was used to detect antibodies to *Toxocara* spp. In the sera. The sensitivity of the ELISA method, based on use of goat anti-mouse polyvalent antibodies, has been validated by several studies on sera of different rodent species, including *M. arvalis* and *M. glareolus*^45–47^. Larval excretory-secretory (E/S) antigen of *T. canis* was prepared as described by De Savigny et al.^48^ and used to detect antibodies to *Toxocara* spp. This antigen has been tested and shown to be specific without cross-reactions with sera of mice experimentally infected with *Toxascaris leonina* and *Ascaris suum*^49^*.*

Microtiter ELISA plates (Nunc; Maxisorp, Denmark) were coated with 100 μl/well antigens diluted in carbonate buffer (pH 9.6) and left standing overnight at 4 °C. The final dilution of antigens was 1 μg protein/ml for E/S *T. canis* antigen. The plates were washed four times with distilled water/0.05% Tween-20 (washing solution). Then 100 μl of the sera, diluted 1:200 in 5% non-fat milk in phosphate buffer (PBS; pH 7.2), were added to the wells and the plates were incubated for 1 h at 37 °C and washed afterwards, as described previously. Next 100 μl of conjugate were added, comprising horse-radish peroxidase-labelled anti-mouse immunoglobulin (Anti-mouse polyvalent immunoglobulins IgG, IgA, IgM; Sigma-Aldrich, Steinheim, Germany) diluted 1:8000 in PBS, and followed by incubation for 1 h at 37 °C and a subsequent washing step. Antibody reactions were visualised by adding 100 μl of the substrate (o-phenylenediamine/methanol diluted 1:100 with 0.05% H_2_O_2_) and the plates were placed in the dark at room temperature. After an incubation period of 20 min, the reaction was subsequently stopped by adding 50 µl of 4 M H_2_SO_4_ and optical densities (OD) were read at 490 nm.

Since no positive control sera from *Toxocara* spp. infected *Microtus* spp. and *M. glareolus* were available, the cut-off value was determined according to Naguleswaran et al.^50^. The first cut-off value was determined by the mean of all sera on the microtiter plate plus three standard deviations (SD). Sera with OD above this value were then excluded, and the remaining sera were used to calculate the mean absorption (Mneg) and the standard deviation (SDneg) of negative samples. Sera with OD values above Mneg + 4 SDneg were considered to be positive.

### Soil samples collection and eggs detection

Since the highest seroprevalence was found in open grassland vole species, we collected soil samples (*n* = 35) in September 2022 from open grasslands located in Urwitałt. We collected 100 g soil samples with vegetation from area where rodent trapping was performed.

Isolation of parasites eggs was preceded by the modified sedimentation–flotation technique from 50 g soil samples collected from open-grasslands. The analyzed samples were homogenized in 400 mL beakers with detergent (Tween-20 solution 0.0025%) for 60 s and set aside for 30 min. The suspension was filtered through a 200-μm sieve to high-capacity centrifuge tubes and centrifuged at 2600*g* for 10 min. After removing of the supernatant, the suspension was homogenized with saturated solution of NaCl and sucrose (specific gravity 1.25 g/mL) and centrifugal flotation at 2600*g* for 2 min was performed. Next microscopic observation was performed using Zeiss Axiolab 5 microscope (Zeiss, Oberkochen, Germany).

### Statistical analysis

Percentage of animals infected (prevalence) is given with 95% confidence limits in parenthesis (CL_95_). We calculated the values using a bespoke software based on the work of Rohlf and Sokal^51^.

The statistical approach has been documented comprehensively in our earlier publications^27,52–54^. For analysis of prevalence, we used maximum likelihood techniques based on log-linear analysis of contingency tables in the software package IBM SPSS Statistics Version 21 (IBM Corporation). This approach is based on categorical values of the factors of interest, which are used to fit hierarchical log-linear models to multidimensional cross-tabulations using an iterative proportional-fitting algorithm and detect associations between the factors, one of which may be the presence/absence of anti-*Toxocara* spp. antibodies. First, we tested whether seroprevalence differed between forest and open grassland vole species. Next, we implemented a full factorial model that incorporated as factors sex (2 levels, males and females), age (3 levels), and species (2 levels, forest and open grassland species). The presence or absence of antibodies against *Toxocara* spp*.* (seroprevalence) was considered a binary factor (0/1). These factors were fitted initially to all models that were evaluated. For each level of analysis, beginning with the most complex model involving all possible main effects and interactions, those combinations that did not contribute significantly to explaining variation were eliminated stepwise, starting with the highest level interaction (backward selection procedure). A minimum sufficient model was then obtained, for which the likelihood ratio of χ^2^ was not significant, indicating that the model was sufficient in explaining the data. The importance of each term in interactions involving seroprevalence in the final model was assessed by the probability that its exclusion would alter the model significantly, and these values are given in the text. We next fitted a model without bank vole data, but with each of the three grassland species as a separate level within the factor “species”, to determine whether seroprevalence differed between the three grassland species. The possible influence of sex and age was then evaluated in a model confined to grassland species but without distinguishing between them. The remaining terms in the final models in each case, that did not include seroprevalence, are not given but can be made available from the authors on request.

### Ethics approval

This study was carried out according to the recommendations in the Guidelines for the Care and Use of Laboratory Animals of the Polish National Ethics Committee for Animal Experimentation. The project was approved by the First Warsaw Local Ethics Committee for Animal Experimentation which also has overarching responsibility for fieldwork involving the trapping and culling of wild vertebrates for scientific purposes (decision no. 148/2011 and 406/2013). The study was performed according to the ARRIVE guidelines 2.0.

## Results

### Seroprevalence analysis

We found anti*-Toxocara* spp. antibodies in the sera of all four rodent species with an overall seroprevalence of 2.8% [1.9–4.1%]. There was a significant difference in seroprevalence between forest and open-grassland species (*χ*^2^_1_ = 28.6; *P* < 0.001), with grassland species (*M. arvalis*, *M. agrestis*, and *A. oeconomus)* showing 16-fold higher seroprevalence (15.7% [8.7–25.9%]) than the forest-dwelling, *M. glareolus* (0.98% [0.5–1.8%]) (Table 1). In this model, there was a significant effect of host sex on seroprevalence (*χ*^2^_1_ = 5.8; *P* = 0.016), with females showing 4.1-fold higher *Toxocara* spp. seroprevalence than males, arising mainly because all five seropositive bank voles were female, and seropositivity was female biased also in *M. arvalis* (Table 1).

**Table 1:** Seroprevalence of *Toxocara* spp. in voles from NE Poland.

	Host age			Host sex		Combined	Seroprevalance % (95% CL)
	1	2	3	Males	Females		
*My. glareolus*	0/138	1/173	4/196	0/251	5/256	5/507	1.0 (0.5–1.8)
*M. arvalis*	0/5	2/9	5/32	1/16	6/30	7/46	15.2 (6.1–31.7)
*A. oeconomus*	0/0	1/2	1/12	2/10	0/4	2/14	14.3 (2.6–42.6)
*M. agrestis*	0/1	1/3	1/6	0/5	2/5	2/10	20.0 (3.7–55.4)
Combined	0/144	5/187	8/246				**2.8 (1.9–4.1)**

The table shows the number of positive animals by sample size in the three age classes, in the sexes and combined, as well as the overall seroprevalence by host species. Host age 1—immature juveniles, host age 2—young adults, host age 3—breeding older animals.

Significant values are in bold.

Four of the seropositive bank voles were in the oldest age class and no juveniles were seropositive, so we did not explore further seroprevalence in bank voles. A model confined to open grassland species showed that seroprevalence did not vary significantly between the three open grassland dwelling species (*χ*^2^_2_ = 0.298, *P* = NS). Therefore, we explored the possibility that age or sex may have affected seroprevalence in grassland species by combining all three into one taxon and found that neither age (*χ*^2^_2_ = 3.64, *P* = 0.162) nor sex (*χ*^2^_1_ = 1.39, *P* = 0.239) affected seroprevalence significantly.

### Soil contamination analysis

**Figure 2:**
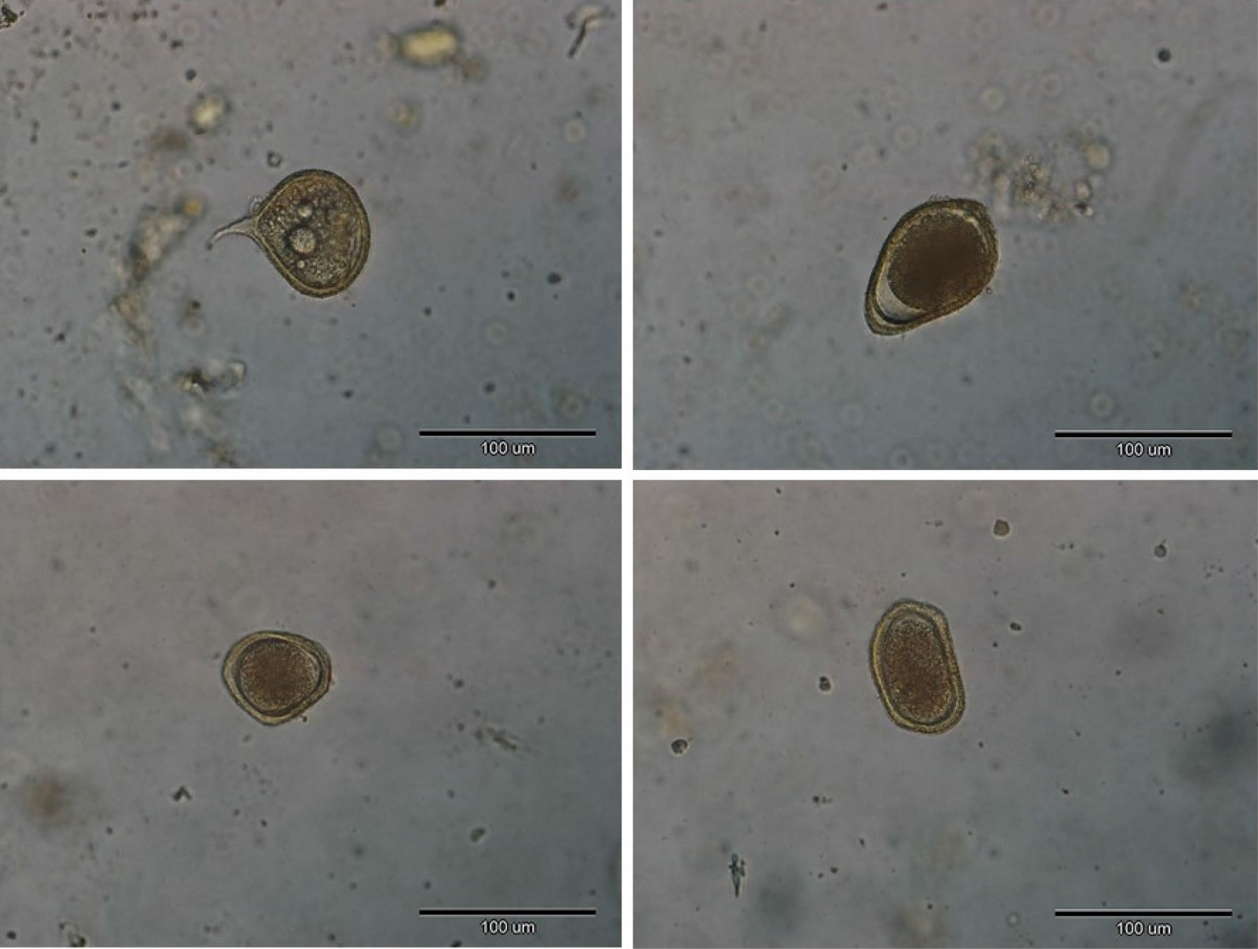
*Toxocara* spp. eggs found in soil collected from open grasslands located in Site 4. Pictures show unembryonated eggs with typical golden colour, spherical to slightly pear shaped, thick-shelled, and a pitted surface. Black bar indicates 100 µm.

We found four out of 35 soil samples collected at Site 4 (Urwitałt open grasslands) being contaminated with *Toxocara* spp. eggs giving prevalence of 11.4% (4.6–24.4). Figure 2 presents recovered *Toxocara* spp. eggs.

## Discussion

Soil-transmitted helminths remain a massive global health problem. Diseases caused by infection with these parasitic worms affect 1.45 billion people each year, mostly among impoverished populations^55^. Environmental contamination with zoonotic pathogens constitutes a significant threat to humans and wild and domestic animals, zoonotic helminth infections being responsible for 1.9 million DALYs (disability-adjusted life years) globally^56^.

In this study, we analysed the seroprevalence of *Toxocara* spp. in sylvatic rodent populations in NE Poland from both open grasslands and neighbouring forests. We confirmed the presence of antibodies against *Toxocara* spp. in all four investigated species resulting in an overall seroprevalence of 2.8%. Our results are in line with other reports from Central Europe, with seroprevalence varying between 2.8 and 15.1%^45,57–61^. The most comprehensive studies assessing the prevalence of *Toxocara* spp. in wild rodent populations have been carried out in Slovakia. Dubinský et al.^11^ examined a total of 582 small mammals from 16 species. Overall, 15.1% were seropositive, with high variability between different species. A strong host species impact has been observed also in other studies carried out in Slovakia, where a seropositivity of 7.7% was reported among 710 rodents^62^, 6.4% among 2140 rodents^45^ and 6.6% among 1523 rodents^28^. Data on the presence of *Toxocara* spp. among rodents in Poland are scarce. Ninety rodents of three species (*Apodemus agrarius*, *A. flavicollis* and *M. glareolus*) from the sub-urban area of Wrocław were tested for *Toxocara* larvae and only *A. agrarius* were found to be positive for *Toxocara*, with prevalence reaching 12.9%^63^. Dvorožňáková et al. carried out a study in Białowieża Primeval Forest, one of the best-preserved lowland primeval forests in Europe, where 2.8% of 106 rodents were seropositive^64^.

We analysed intrinsic factors (host species, host age and host sex) to assess their effects on the seroprevalence of *Toxocara* spp. We found a strong impact of host species on *Toxocara* seroprevalence, with a significantly higher seroprevalence among open-grassland host species relative to forest-dwelling species. Our results indicate that open-grasslands are more contaminated with *Toxocara* spp. than forests. We confirmed the contamination by the presence of anti-*Toxocara* spp. antibodies in rodents and eggs in the subsequent analysis of soil samples. In contrast to our study, previous studies have compared seroprevalence between urban, suburban and rural sites, and in these the prevalence of *Toxocara* has been shown to be highly dependent on the level of urbanisation of the sampling localisation. For example, Dubinský et al.^11^ observed that synanthropic and hemisynantropic rodents were more frequently seropositive (25–32%) than sylvatic rodents (6.2–11.3%), while Reperant et al.^47^ found that seroprevalence among small rodents was higher in urban (13.2%) than peri-urban (3.3%) and rural areas (4.9%). Data from Poland are consistent with this hypothesis, with suburban areas being more contaminated than well-preserved rural environments^63,64^.

*Toxocara* eggs can survive for years in soil, constituting a source of infection for paratenic hosts^65^. Small mammals can become infected with *T. canis* when they ingest infective eggs shed by dogs (*Canis lupus familiaris*), red foxes (*Vulpes vulpes*), racoon dogs (*Nyctereutes procyonoides*) or wolves (*Canis lupus*). Examination of wolf scats for the presence of *Toxocara* eggs has revealed a prevalence of 13.5%^66,67^ and 15.1% seroprevalence has been reported in racoon dogs in NE Poland^68^. Although wolves and racoon dogs may contaminate the environment with *Toxocara* eggs, their role is likely less important than that of dogs and red foxes. The latter two species are considered the primary source of environmental contamination with *Toxocara* eggs^13^, and a study conducted by Cisek et al. suggests that *T. canis* is common in domestic dogs (2.67–55%) and red foxes (43%) in NE Poland^69^. However, free-roaming, not-dewormed stray dogs may also be a source of infection and data show that they are more frequently infected with *T. canis* than domestic dogs^70^. An anthropogenic environment therefore facilitates *Toxocara* transmission due to the high density of canids, mostly kept as pets.

Our grassland study site was a previously cultivated field that is exposed to stray and pet dogs from visitors to the region and the inhabitants of the neighbouring town, and to the local fox population. A study performed in NE Poland showed that *Microtus* spp. were found in 73% of red fox stomachs and constituted 47% of their consumed food volume^71^. It is thought that places with a higher prey density are more frequently inhabited by foxes resulting in an accumulation of faeces with *Toxocara* eggs^62^. The abundance of *Microtus* spp. on our grassland sites may explain why foxes inhabit this locality frequently, leading to soil contamination and thereby infection of grassland rodents.

We also studied the impact of intrinsic factors such as age and sex of the host on the presence of *Toxocara* spp. antibodies. Our previous studies have reported differences in seroprevalence and prevalence between males and females in other rodent-borne parasites^27,72,73^, but here no consistent difference between the sexes was detected. While seroprevalence appeared to be female biased in a model that included bank voles and the grassland species (the latter as one taxon), this arose mainly because all five infected bank voles were female and there was a trend for female bias among *M. arvalis* and *M. agrestis*. However, when we combined all grassland species into one taxon and excluded bank voles, no sex bias was evident among the grassland species. This finding is consistent with results from other studies^28,47^, suggesting no sex bias in *Toxocara* infections in rodents. Perhaps surprisingly, we found no significant impact of host age on *Toxocara* seroprevalence. Our previous reports on seroprevalence of other zoonotic nematodes, i.e. *Trichinella spiralis,* in the same population of rodents, showed that seroprevalence increases with host age^72^, a finding that is consistent with the idea that the likelihood of accumulating pathogens and antibodies against those pathogens increases with host age. However, no such age effect was observed in the present study. Recently Maciag et al. highlighted the problem of reliable, unambiguous differentiation between *T. canis* and *T. cati*, another zoonotic *Toxocara* species whose definitive hosts are felines^74^. Research has been asymmetrically focused on *T. canis* while neglecting *T. cati*. It is important to note that due to homology between TES (*Toxocara* excretory-secretory) antigens, cross-reactivity between *T. canis* and *T. cati* may occur in antibody assays. Due to this diagnostic limitation, the seroprevalence of *T. cati* is not known. There are significant differences in the behaviour of domestic cats and dogs. Domestic cats often move unrestrained and are allowed to roam freely in neighbourhoods^75^ where they prey on birds and rodents and are likely to eat paratenic hosts infected with *Toxocara*^76^*.* Cats may therefore have a vital role in the circulation of *Toxocara* spp.

At the time of our study we did not collect rodent brain samples to perform search for *Toxocara* spp. larvae for molecular diagnostics to distinguish between the infection by *T. canis* or *T. cati*. To the best of our knowledge, only one study has been published to-date in which molecular techniques were used to access and differentiate between the prevalence of *T. canis* and *T. cati* in wild rodents. It found that 3.1% and 1.6% of rodents were infected with *T. canis* and *T. cati,* respectively^77^. However, the differences in life cycle of *T. canis* and *T. cati* do not impact development of *Toxocara* nematodes in paratenic hosts. Another limitation of our study was small number of open grassland species (*M. arvalis*, *M. agrestis*, and *A. oeconomus*) comparing to the forest-dwelling, *M. glareolus*. Field studies in rodent populations are always unpredictable in terms of number of collected individuals. This is caused by rodent seasonal cycles, food resources, and other intrinsic and extrinsic factors^22^.

Small mammals serve as a significant reservoir of *Toxocara* larvae, which can survive in their tissues for many months^78^. It is thought that rodents support the parasite's survival especially during unfavorable conditions such as during periods when there is an absence of definitive hosts^65,79,80^. The high prevalence of *Toxocara* larvae in paratenic hosts may lead to spillover from the sylvatic to the synanthropic cycle due to stray and pet dogs feeding on wild rodents^81^. Biomonitoring of pathogen dynamics in their wildlife reservoir is pivotal in understanding their epidemiology and facilitating informed decisions on the control of zoonotic diseases^32,82,83^. Our study shows that different environments may differ significantly in supporting the local presence of the parasite, and hence sampling design is of critical importance in studies of the regional prevalence of parasites. We conclude that seroprevalence of *Toxocara* in wild rodents is a good indicator of environment contamination with *Toxocara* spp. and therefore may constitute a more direct measure for assessment of environment contamination with the infective stages of this nematode than soil samples (Supplementary information S1).

## Supplementary Information

Supplementary Information.

## Data Availability

All data generated or analysed during this study are included in this published article (and its supplementary information files).
