# Publisher Correction: Grassland versus forest dwelling rodents as indicators of environmental contamination with the zoonotic nematode *Toxocara* spp.

**DOI:** 10.1038/s41598-023-30420-6

**Published:** 2023-03-06

**Authors:** Martyna Krupińska, Daniela Antolová, Katarzyna Tołkacz, Klaudiusz Szczepaniak, Aneta Strachecka, Aleksander Goll, Joanna Nowicka, Karolina Baranowicz, Anna Bajer, Jerzy M. Behnke, Maciej Grzybek

**Affiliations:** 1 Department of Tropical Parasitology, Medical University of Gdansk, Powstania Styczniowego 9B, 81‑519 Gdynia, Poland; 2 Institute of Parasitology of SAS, Košice, Slovakia; 3 University of Warsaw, Warsaw, Poland; 4 Institute of Biochemistry and Biophysics, Polish Academy of Science, Warsaw, Poland; 5 University of Life Sciences in Lublin, Lublin, Poland; 6 University of Nottingham, Nottingham, UK

Correction to: *Scientific Reports* 10.1038/s41598-022-23891-6, published online 10 January 2023

In the original version of this Article, Klaudiusz Szczepaniak was incorrectly listed as a corresponding author. The correct corresponding author for this Article is Maciej Grzybek. Correspondence and request for materials should be addressed to maciej.grzybek@gumed.edu.pl.

The original Article has been corrected.

